# TMS affects moral judgment, showing the role of DLPFC and TPJ in cognitive and emotional processing

**DOI:** 10.3389/fnins.2014.00018

**Published:** 2014-02-13

**Authors:** Danique Jeurissen, Alexander T. Sack, Alard Roebroeck, Brian E. Russ, Alvaro Pascual-Leone

**Affiliations:** ^1^Berenson-Allen Center for Noninvasive Brain Stimulation, Beth Israel Deaconess Medical Center, Harvard Medical SchoolBoston, MA, USA; ^2^Department of Cognitive Neuroscience, Faculty of Psychology and Neuroscience, Maastricht UniversityMaastricht, Netherlands; ^3^Department of Vision and Cognition, Netherlands Institute for Neuroscience, Royal Netherlands Academy of Arts and SciencesAmsterdam, Netherlands; ^4^Maastricht Brain Imaging CenterMaastricht, Netherlands; ^5^Department of Psychology, Harvard UniversityCambridge, MA, USA

**Keywords:** morality, decision-making, TMS, emotion and reason

## Abstract

Decision-making involves a complex interplay of emotional responses and reasoning processes. In this study, we use TMS to explore the neurobiological substrates of moral decisions in humans. To examining the effects of TMS on the outcome of a moral-decision, we compare the decision outcome of *moral-personal* and *moral-impersonal* dilemmas to each other and examine the differential effects of applying TMS over the right DLPFC or right TPJ. In this comparison, we find that the TMS-induced disruption of the DLPFC during the decision process, affects the outcome of the moral-personal judgment, while TMS-induced disruption of TPJ affects only moral-impersonal conditions. In other words, we find a double-dissociation between DLPFC and TPJ in the outcome of a moral decision. Furthermore, we find that TMS-induced disruption of the DLPFC during non-moral, moral-impersonal, and moral-personal decisions lead to lower ratings of regret about the decision. Our results are in line with the dual-process theory and suggest a role for both the emotional response and cognitive reasoning process in moral judgment. Both the emotional and cognitive processes were shown to be involved in the decision outcome.

## Introduction

In the case of the so-called *trolley* or *switch dilemma* (Thomson, 1986), most people find it appropriate to turn a switch, which changes the direction of a train, to save the lives of five workmen at the expense of the life of one. By contrast, in the case of the *footbridge dilemma*, most people argue that it is inappropriate to push a stranger in front of a train, even if it can save the lives of five. Although the question in both scenarios is similar (is it appropriate to save the lives of five at the cost of one other?), the agents' responses tend to be different (Greene et al., 2001). Moral dilemmas occur in situations in which it is unclear which decisions are right and wrong based on one's moral values. Moral dilemmas are categorized as moral-impersonal (e.g., trolley or switch dilemma) and moral-personal dilemmas (e.g., footbridge dilemma) (Greene et al., 2001). Although the examples above are exceptional situations; in our social live, at work, or in other situations we may encounter, we often have to decide how we will handle a situation in such a way that is morally acceptable. The current study aims at improving our understanding of how these moral decisions are made by the human brain.

The dual-process theory tries to explain these differences (Greene et al., 2001, 2004). According to this theory, moral decision-making involves an automatic emotional response and a controlled application of a utilitarian decision-rule. The thought of being responsible for the death of another person elicits an aversive emotional response, but in parallel, cognitive reasoning processes favors the utilitarian option. In moral-personal dilemmas, this emotional response is thought to be too strong to be overruled by the cognitive system. Hence, while one might agree with the utilitarian standpoint that it is appropriate to save the lives of five at the cost of one other, being the one that actually physically takes the other life is too high of a threshold. In contrast, in moral-impersonal dilemmas, cognitive control over the lower emotional response leads participants to favor the utilitarian option (Greene, 2007).

In previous lesion and neuroimaging studies, the dorsolateral prefrontal cortex (DLPFC) and temporal-parietal junction (TPJ), among other areas, were found to be involved in moral decision-making (Greene et al., 2001, 2004; Heekeren et al., 2005; Mendez et al., 2005; Ciaramelli et al., 2007; Koenigs et al., 2007, 2008; Moll and de Oliveira-Souza, 2007; Young and Koenigs, 2007; Kahane and Shackel, 2008; Prehn et al., 2008; Cushman et al., 2012). Decision making, reward processing, risk taking, and social cognition are often associated with processes in the DLPFC (e.g., Yamasaki et al., 2002; Forbes and Grafman, 2010; Mullette-Gillman et al., 2011; Essex et al., 2012; Hutcherson et al., 2012; Minati et al., 2012; Sokol-Hessner et al., 2012; Soo Cho et al., 2012; Steinbeis et al., 2012). Neurons in the prefrontal cortex were found to be involved in cost-benefit analysis and categorize stimuli based on the predicted consequences (Hosokawa et al., 2013). The connectivity between relevant brain areas can depend upon the specific task (Baumgartner et al., 2012). In a study using transcranial alternating current stimulation, Sela et al. (2012) found that DLPFC activity is important for adaptive decision-making is a risk-taking situation.

Related to moral decision-making is theory-of-mind (TOM) and empathy, which are often associated with TPJ functioning (Saxe, 2006; Young et al., 2010). TOM is a cognitive mechanism which is used when one tries to understand and explain the knowledge, beliefs, and intention of others (Young et al., 2010; Korkmaz, 2011). ToM develops at an early age in developing children and at the age of around 3–4 years, children can use a ToM in their behavior (Korkmaz, 2011). Moral decision making and ToM are closely related to each other, i.e., while making moral decisions, people will often place themselves in the position of the “decision-maker” or the ‘victim’ in the dilemma. ToM may also lead to a shared emotional response with the described character in the moral dilemma. TPJ plays an important role in ToM, empathy, and social cognition (Saxe and Kanwisher, 2003; Carter et al., 2012; Santiesteban et al., 2012; Van Overwalle and Vandekerckhove, 2013). Young et al. (2010) have shown that when the right TPJ is disrupted by magnetic stimulation, the role of beliefs in moral judgments is reduced.

The emotional and cognitive reasoning processes as identified in the dual-process theory have been related to activity in the DLPFC and TPJ (see Greene et al., 2001, 2004 for fMRI results in which the non-moral, moral impersonal, and moral personal dilemmas were tested to identify the brain areas in which activity is related to moral decision-making). Specifically, the activity in the prefrontal cortex is thought to be important for the cognitive reasoning process, which can counteract the emotional response. Greene et al. (2001) found that the medial portions of the medial frontal gyrus, the posterior cingulate gyrus, and the bilateral angular gyrus showed a higher BOLD response in the moral-personal condition than the moral-impersonal condition. The right middle frontal gyrus and the bilateral parietal lobes showed a lower BOLD response in the moral-personal condition than in the moral impersonal and non-moral condition. These results were very similar to the results presented by Greene et al. (2004). Furthermore, Greene et al. (2004) show an increased BOLD response for the bilateral amygdale in personal compared to the impersonal dilemmas.

Increased activity in DLPFC is thought to be related to the increased cognitive effort that is put in counteracting the (non-utilitarian) emotional response (Greene et al., 2004). Given the role of the prefrontal cortex in moral decision-making, we hypothesize that when magnetically stimulating this area in our experiment, we will selectively influence the decision process of the moral personal dilemmas because the cognitive reasoning for which the DLPFC is important is disrupted. The other component of the dual processing in moral decision-making is the emotional response. Because the activity in the TPJ is related to emotional processing and theory of mind (Saxe and Kanwisher, 2003; Young et al., 2010), and because the activity in the parietal cortex is increased for moral-impersonal dilemmas compared to moral-personal questions (Greene et al., 2004), we suspected an important role for this area in moral cognition. More specifically, considering the data from fMRI experiments in moral cognition, we hypothesized that when magnetically stimulating this area during a moral decision, this will selectively influence the decision process of moral-impersonal dilemmas.

We designed a TMS experiment to examine whether the DLPFC and TPJ are indeed differentially involved in moral-judgment. Whereas lesions are often not focal, patients may develop compensatory strategies, and results across patients are difficult to compare, our TMS design does not suffer from these disadvantages. Additionally, whereas neuroimaging correlates activity to behavior, TMS allows us to establish a causal link between the two. To investigate this role of DLPFC and TPJ we used chronometric TMS to target the two brain regions and investigate the role of these areas in moral-judgment. Based on the literature about the function of the DLPFC and TPJ, we hypothesized that the DLPFC and TPJ are both involved in moral decision-making. However, because of their different functions, these two areas are expected to each play their own role in moral decision-making. If the DLPFC and TPJ are involved in the moral decision-making process, applying magnetic stimulation over these areas can influence behavioral measures such as reaction time, decision outcome, and evaluation of the decision. To examine the roles of DLPFC and TPJ in emotional processing and cognitive control, we test how they are involved in moral personal and moral impersonal dilemmas. If the DLPFC and TPJ are differentially involved in these two categories of dilemmas, magnetic stimulation will be able to reveal a double-dissociation between these two conditions when stimulating the DLPFC or TPJ.

## Methods

The current study is a Talairach-based MRI-neuronavigated, event-related chronometric TMS study (*N* = 17) aimed at disrupting the activities in the DLPFC or the TPJ (between-subject factor) to examine the behavioral relevance of these areas. We tested the moral-personal, moral-impersonal, and non-moral dilemmas. For further details on the distinction between moral conditions, see Greene et al. (2001). In summary, the moral personal dilemmas are dilemmas in which the described action “(a) could reasonably be expected to lead to serious bodily harm (b) to a particular person or a member or members of a particular group of people (c) where this harm is not the result of deflecting an existing threat onto a different party” (quoted from page 2107 of Greene et al., 2001). The moral impersonal dilemmas only meet one or two of these three criteria. Non-moral dilemmas are related to daily-life situations and do not require moral reasoning nor are they emotionally salient (in other words, they do not fulfill any of the three requirements). The questionnaire is designed such that participants respond “appropriate” and “inappropriate” equally often and has been validated in earlier experiments (the entire questionnaire is available in the supplementary material of Greene et al., 2001).

### Participants

A total of 17 participants (8 males, 9 females; mean age: 23.7 years, range: 18–54 years) were enrolled in this experiment. All participants were healthy volunteers that met the criteria for participating in a TMS experiment. Participants were right-handed, had normal or corrected-to-normal vision, and signed informed consent before the start of the experiment. Participants received monetary compensation for participation in the experiment. One participant was excluded from further analysis because of very short reaction times, (i.e., most responses were given before TMS was applied).

### Conditions and stimuli

Dilemmas were formulated and tested in earlier studies by Greene et al. (2001). Three moral conditions were used in the current experiments: non-moral (control condition, 20 trials), moral-impersonal (19 trials), and moral-personal dilemmas (25 trials), resulting in a total number of 64 trials. Dilemmas were presented in three screens: the first two screens explaining the context, and the third screen asked the question whether a given action is appropriate or inappropriate and reminded the participant which button to press to indicate their answer. The participants read the dilemma at their own pace and by pressing a button they continued to the next screen. Both the response (appropriate or inappropriate) and the reaction times (RT, measured from the onset of the third screen) were recorded. After the participant's response, a fixation cross was presented for 6 s. This fixation period was followed by four consecutive evaluation questions, which the participants rated on a scale from 1 to 6:

- Question 1: How confident are you that you made the right decision? (With 1, not at all confident to 6, very confident).
- Question 2: Do you feel responsible for the outcome of the decision? (With 1, no to 6, yes).
- Question 3: Do you feel regret about the decision? (With 1, no to 6, yes).
- Question 4: Looking back at your decision: would you like to change your answer? (With 1, no to 6, yes).

**Figure 1:**
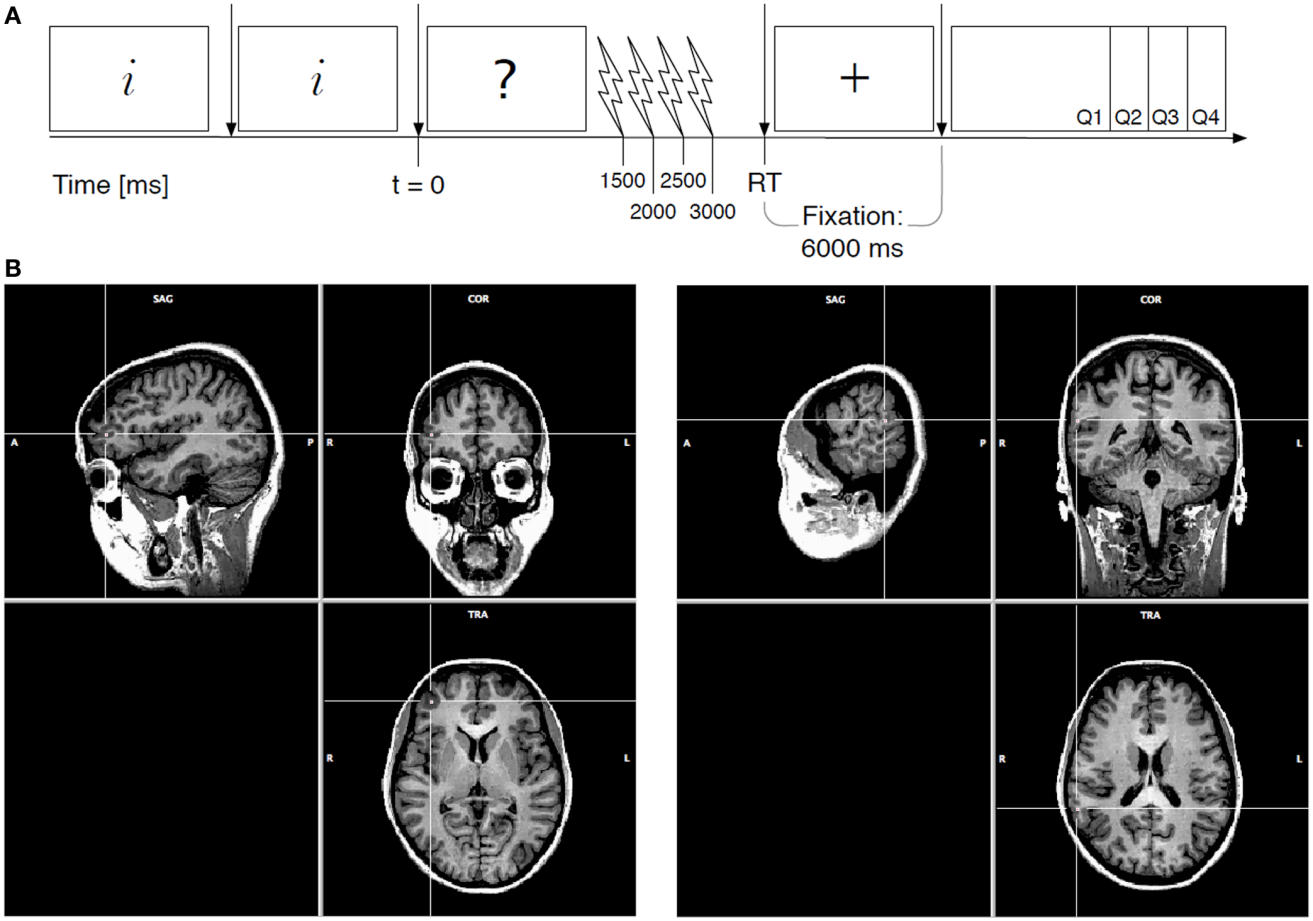
**Schematic overview of a single trial and stimulation areas. (A)** The first two screens provide the participant with information about the dilemma, the third screen asks the question to which the participant responds “appropriate” or “inappropriate.” Reaction time is measured from the onset of the third screen. The button press to indicate the answer is followed by a fixation period and four evaluation questions. **(B)** Stimulation areas. An fMRI image of the three planes through the Talairach point for the DLPFC on the left, and the TPJ on the right (BrainTutor software, BrainVoyager QX).

Based on the literature the right DLPFC and TPJ were selected as target regions for the TMS experiment. In each participant, magnetic stimulation was applied over only one of these two target sites: right DLPFC or right TPJ. After reading the dilemma and continuing to the third screen, a train of three TMS pulses was applied to the target site. The three pulses were spaced 150 ms apart, aiming to disrupt neural activity in the target site for roughly half a second. In order to find the crucial time point at which these three areas are involved in moral judgment, different time points of TMS application were tested as a within-subject factor. The first pulse was applied after 1.5, 2, 2.5, or 3 s after onset of the third screen. The presentation order of the different moral conditions and the time point of stimulation were both randomized for each participant. See Figure 1 for a schematic overview of a single trial.

### Procedure

The participants were instructed that they would be presented with different (moral) dilemmas and that they had to judge whether they would find the described action appropriate or inappropriate if they were to encounter such a situation.

Participants underwent a structural MRI for neuronavigation purposes. Three test pulses were given to the target site for the participant to become familiar with the triple-pulse stimulation. The 64 dilemmas were divided over four separate runs so that the participant could take a break between runs. The stimuli were presented and TMS pulses were externally triggered by Presentation software (Neurobehavioral Systems, San Francisco, CA).

### TMS parameters

TMS was applied using a Magstim Rapid TMS machine and a figure-of-eight TMS coil. Based on preliminary fMRI results (not included in this manuscript) we localized the right DLPFC and right TPJ at the following Talairach coordinates: right DLPFC *x* = +39, *y* = +47, *z* = +7 (see Figure 1B, left); and right TPJ *x* = +60, *y* = −40, *z* = +19 (see Figure 1B, right). Although the TMS target point for the DLPFC is not exactly the same as the coordinates found for the frontal regions by Greene et al. (2001, 2004), we chose this area because our preliminary fMRI results showed a difference in BOLD response between the moral personal and the moral impersonal dilemmas. For each individual participant, the individual anatomical MRI scan was transformed to Talairach-space, then the target areas were defined, and finally the image was transformed back to the native-space of the participant to guide the neuronavigated TMS. The figure-of-eight TMS coil was placed tangential to the scalp and oriented at 90° to the individuals central sulcus for the right DLPFC, and at 45° to the individuals central sulcus for the right TPJ target site with the handle always pointing posteriorly. The position of the TMS coil with respect to the TMS target-areas were monitored online using Brainsight neuronavigation software (version 2.0, Rogue Research). The train of three pulses, 150 ms apart, was applied at 70 percent machine output.

### Data analysis

All the trials in which reaction times were too short (<3.3 s) or too long (>20 s) were removed from the data. Reaction times below 3.3 s were considered to be too short because on these trials the TMS pulses were not applied for the last time condition. To statistically test the effect of stimulation site (right DLPFC and right TPJ) and time condition (1.5, 2, 2.5, and 3 s after onset of the question) on the subjects' response (appropriate or inappropriate), the personal minus impersonal difference score was calculated for each brain area and each time point. This score indicates the moral-condition specific change in the dependent variable for each time point (within subjects) and for each stimulation site (between subjects). We used a *t*-test for each time condition to evaluate whether there were any differences between conditions. Furthermore, the RT and evaluation of the decision (confidence, responsibility, regret, and whether or not the subject would like to change his/her answer) were analyzed. Because there were no time specific effects of TMS on the RT and evaluation of the decision, the data was collapsed over the four different time conditions for each moral condition, and repeated-measures ANOVA was used to test the TMS effect that is specific for a brain area but not time point. When the ANOVA showed a significant result, a paired samples *t*-test was used for *post-hoc* comparisons between conditions.

## Results

### Reaction times

**Figure 2:**
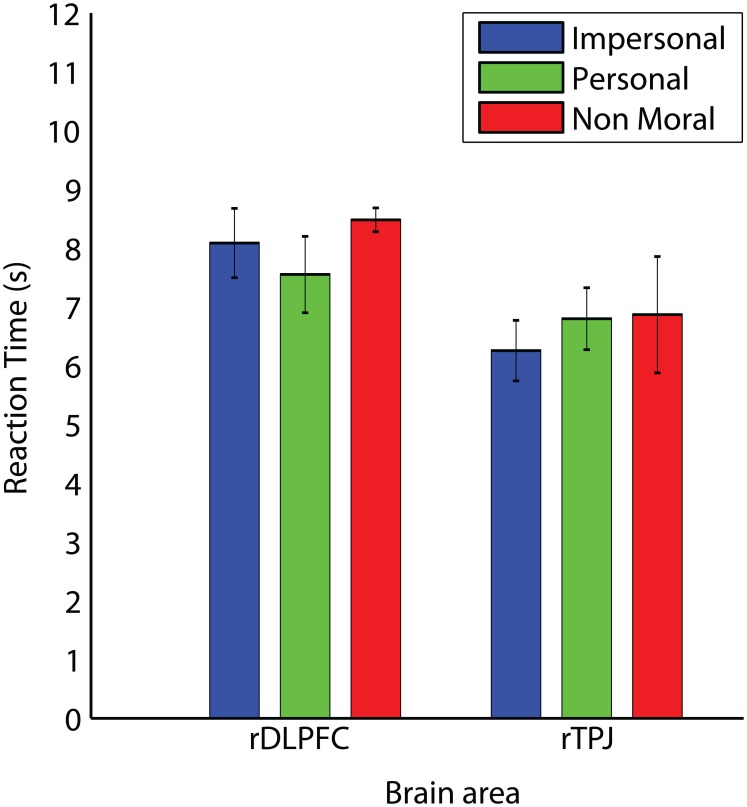
**Reaction times in TMS experiment**. The three different moral conditions grouped over the four different time points of TMS application over the right DLPFC and right TPJ. Error bars indicate one standard error from the mean on both sides.

TMS did not exert an effect on reaction time (*F* = 2.549, *p* = n.s.). Figure 2 shows the reaction time for the different moral conditions for each of the two brain areas (collapsed over time-points).

### Response

**Figure 3:**
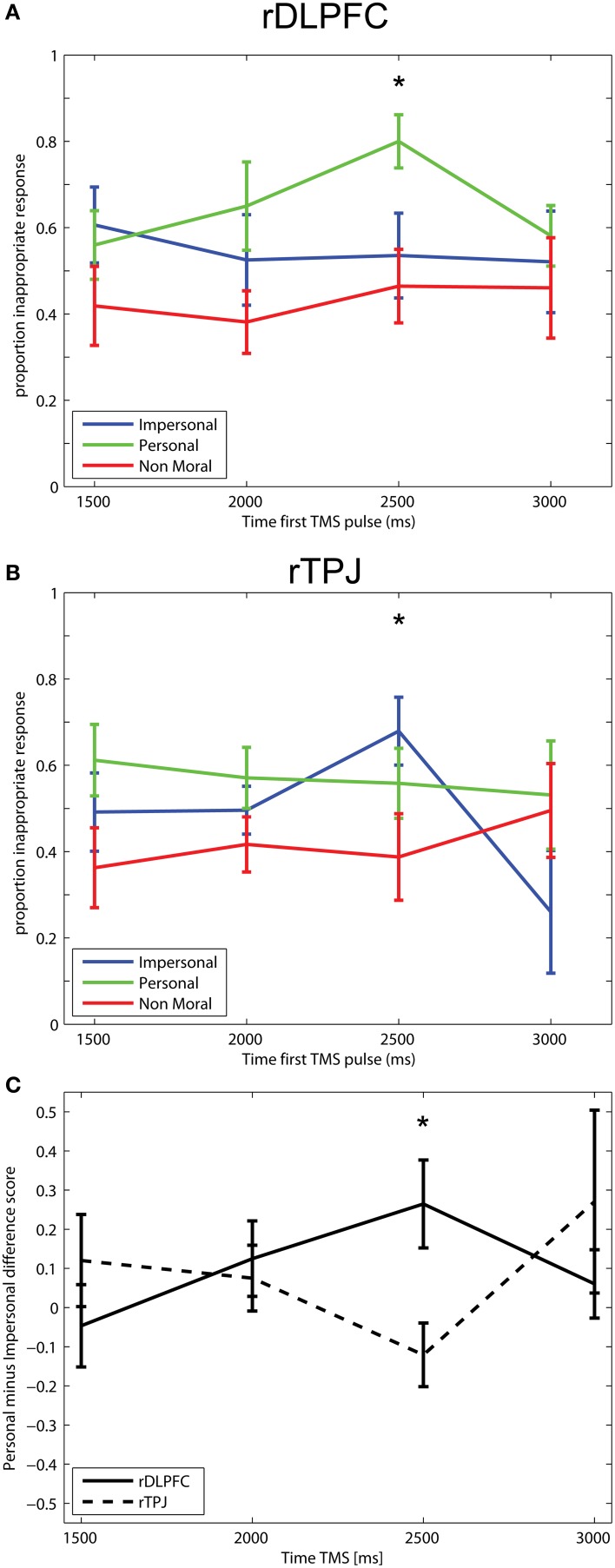
**Responses in TMS experiment**. Proportion inappropriate responses in the three different moral conditions for the four different time points of TMS application. Error bars indicate one standard error from the mean on both sides. **(A)** Right DLPFC—Moral-personal judgment is significantly modulated by TMS over the right DLPFC if it is applied at 2.5 s after onset of the moral question. **(B)** Right TPJ—Moral-impersonal judgment is significantly modulated by TMS over the right TPJ if it is applied at 2.5 s after onset of the moral question. **(C)** Difference between the effects for the right DLPFC vs. the effects of right TPJ. ^*^Indicates a significant difference between the DLPFC and TPJ, *p* < 0.05.

The statistical test of the within-subjects contrast reveals a time-specific double dissociation between the two stimulation sites (DLPFC and TPJ) for the moral-personal and moral-impersonal judgment as shown by a significant difference between the two stimulation sites when stimulated at 2.5 s (*t* = 2.779, *p* < 0.02), and not for the other three time points (*t* = −1.056, *t* = 0.391, and *t* = −0.843, with all *p*-values not significant, for respectively, 1.5, 2, and 3 s). In other words, the number of inappropriate responses is higher in the moral-personal condition if TMS is applied after 2.5 s over the DLPFC, whereas, if the TPJ is stimulated 2.5 s after onset of the moral question the number of inappropriate responses is higher in the moral-impersonal condition. Figure 3 shows the proportion of inappropriate responses for the three moral conditions for, respectively, the DLPFC (Figure 3A) and TPJ (Figure 3B) stimulation, and the difference between these two conditions (Figure 3C). As shown in Figure 3A, the application of TMS on the four different time points over the DLPFC has no effect on the proportion appropriate and inappropriate responses in the moral-impersonal and non-moral judgment. However, TMS over the DLPFC affects the judgment in the moral-personal conditions in a time-specific manner. Where the proportion of inappropriate responses is stable around 60% at TMS time-points 1.5, 2, and 3 s., the number of inappropriate responses is higher, around 80 percent, in the condition were the three TMS pulses were applied 2.5 s after onset of the question on the screen. Figure 3B shows the effect of magnetic stimulation of the rTPJ on moral decision-making. Stimulation of the TPJ does not affect the moral-personal and non-moral judgment (horizontal, parallel lines) but the magnetic stimulation affects the moral-impersonal judgment in a time-specific manner. The proportion of appropriate and inappropriate responses is changed in late time-points. Figure 3C shows the differential effect of the magnetic stimulation over the DLPFC vs. TPJ.

After establishing the time-specific double-dissociation in the first analysis, which shows that TMS over DLPFC specifically affects the outcome of the moral-personal dilemmas, and TMS over TPJ specifically affects the outcome of the moral-impersonal dilemmas, we ran additional *post-hoc* comparisons to confirm this result. These *post-hoc* comparisons aimed at further examining the effect when the moral dilemmas are compared to the non-moral control condition. First of all, we used a paired-samples *t*-test to compare the moral personal vs. the non-moral condition at time point 2.5 for the DLPFC. This *t*-test shows a significant difference between the moral personal and the non-moral condition (*t* = −2.890, *p* = 0.02). Second, we used a paired-samples *t*-test to compare the moral impersonal vs. the non-moral condition at time point 2.5 for the TPJ. This *t*-test shows a trend for the difference between the moral impersonal and non-moral condition (*t* = 2.159, *p* = 0.07). To further examine the specificity of the effect, we compared the moral personal dilemmas to the moral impersonal dilemmas at time point 2.5. For the DLPFC this yielded a significant result (*t* = 2.355, *p* = 0.05), for the TPJ, this result is not significant (*t* = −1.486, *p* = n.s.).

Additionally, we have performed further *post-hoc* comparisons to examine the specificity of the time-point. We used paired-samples *t*-tests to compare the outcome of the decision at trials in which TMS was applied at 2.5 s to the outcome of the decision at trials in which TMS was applied at 1.5, 2, and 3 s. For the DLFPC, this comparison was done for the personal dilemmas. The comparison of time point 2.5 against the 1.5 and 3 s time point is significant (*t* = −2.890, *p* = 0.02 for time point 1.5, and *t* = −2.377, *p* = 0.05 for time point 3). The difference between time point 2.5 and 2 is less pronounced (*t* = −1.810, *p* = n.s.). This may be caused by some intersubject variability where the exact time point may be around the 2.5 s time-point. For the TPJ data, this comparison was done for the impersonal dilemmas. The comparison of the 2.5 time point against the 2 and 3 s time point is significant (*t* = −2.982, *p* = 0.02) and close to significant (*t* = −2.283, *p* = 0.06), respectively. The comparison of the 2.5 and 1.5 is not significant (*t* = −1.488, *p* = n.s.) and the effect in the TPJ data is therefore less clear. The time-point comparison shows a clearer result for the DLPFC than it does for the TPJ.

Given these *post-hoc* tests, the effect in the first analysis is driven by both the effect of TMS on the DLPFC as well as the TMS on the TPJ and supports the conclusion for before mentioned double dissociation. However, the results for the DLPFC seem to be statistically more reliable than the results for the TPJ.

### Evaluation

In order to test for a change in the evaluation of the decision outcome, repeated measures ANOVAs were performed for the ratings on the four evaluation questions. Overall, the participants were very confident about their decisions and they rated their level of confidence on average between four and six for all three moral conditions and two stimulation sites. However, there was a main effect of moral condition (Greenhouse-Geisser corrected, *F* = 41.793, *p* < 0.01). The *post-hoc* paired samples *t*-test showed that all three conditions differed significantly from each other (impersonal vs. personal: *t* = 3.185, *p* < 0.01; impersonal vs. non-moral: *t* = −6.439, *p* < 0.01; personal vs. non-moral: *t* = −8.317, *p* < 0.01). Participants showed the highest confidence levels for non-moral decisions (average rating of 5.65), followed by their confidence in the moral-impersonal condition (average rating of 5.10), finally participants showed the lowest confidence levels in the moral-personal condition (average rating of 4.78). There was no effect of TMS on the confidence rating (*F* = 3.406, *p* = n.s.).

In all moral conditions, participants felt very responsible for the decision that they made with ratings that were on average 5.30. There were no differences between conditions: no effect of TMS (*F* = 0.053, *p* = n.s.) and no effect of moral condition (*F* = 0.754, *p* = n.s.).

**Figure 4:**
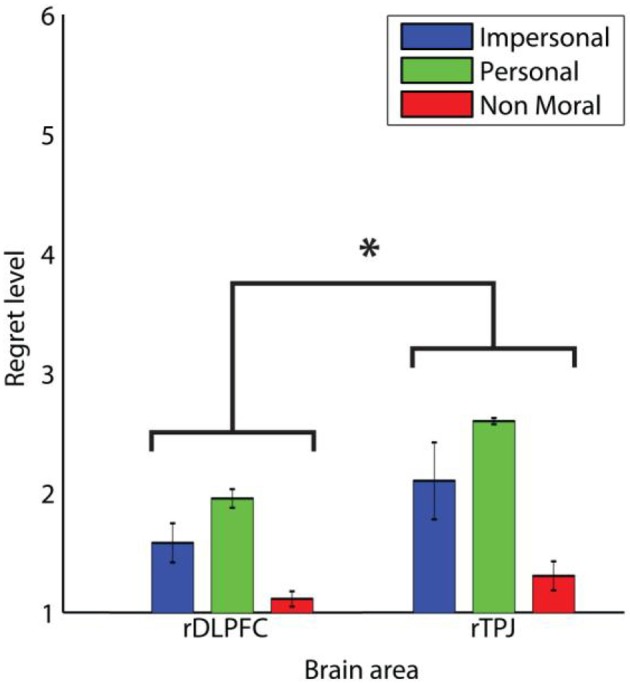
**Regret levels in TMS experiment**. The three different moral conditions are grouped over the four different time points of TMS application over the right DLPFC and right TPJ. Error bars indicate one standard error from the mean on both sides. ^*^Indicates a significant difference between the DLPFC and TPJ, *p* < 0.05.

The regret ratings were influenced by magnetic stimulation. Figure 4 shows the regret ratings for the three moral conditions and the two different stimulation sites. The main effect of moral condition was significant (Greenhouse-Geisser corrected, *F* = 39.960, *p* < 0.01) and *post-hoc* paired samples *t*-test comparisons show that all three moral conditions differ significantly from each other (impersonal vs. personal: *t* = −3.832, *p* < 0.01; impersonal vs. non-moral: *t* = 6.278, *p* < 0.01; personal vs. non-moral: *t* = 7.001, *p* < 0.01). The highest regret rating was observed in the moral-personal dilemmas, lower ratings were observed in the moral-impersonal dilemmas, and very low ratings were observed in the non-moral control questions. The main effect of stimulation site was significant (*F* = 7.390, *p* < 0.02) and the pairwise comparisons between the two brain sites showed that the regret ratings after TMS over the right DLPFC are lower from the regret ratings after magnetic stimulation of the right TPJ.

For the last question, participants indicate that they do not wish to change their judgment. There is no effect of TMS (*F* = 4.062, *p* = n.s.) but the main effect of moral condition is significant (Greenhouse-Geisser corrected, *F* = 17.970, *p* < 0.01). The paired samples *t*-test was used for *post-hoc* comparisons to show that this effect is caused by higher ratings in the moral-personal (average rating of 1.50) and moral-impersonal dilemmas (average rating of 1.40) compared to the non-moral dilemmas (average rating of 1.17) (impersonal vs. personal: *t* = −1.747, *p* = n.s.; impersonal vs. non-moral: *t* = 3.927, *p* < 0.01; personal vs. non-moral: *t* = 5.221, *p* < 0.01).

## Discussion

Previous research has shown that the DLPFC plays a role in reasoning and decision-making (Baraclough et al., 2004; Ernst and Paulus, 2005; Van't Wout et al., 2005; Fleck et al., 2006). Our results confirm this role of DLPFC in moral decision-making. (Greene et al., 2001, 2004) show a stronger activity in the frontal cortex for the moral-personal condition than in the non-moral and moral-impersonal condition. They hypothesize that this is because the high-conflict moral-personal dilemmas require more cognitive control over the initial emotional response. In moral-personal dilemmas, the emotional response generally favors the non-utilitarian option. The cognitive reasoning process can overrule the initial emotional response and thereby favor the utilitarian option. In other words, if the emotional response does not reach a certain “threshold,” the cognitive reasoning can overcome the initial emotional response. This threshold determines whether or not one would sacrifice one thing in order to achieve a pre-set goal. For example, in the footbridge and trolley dilemmas, the participant has to decide whether or not it is appropriate to sacrifice one life in order to save five. The dual-process theory (Greene et al., 2001, 2004) predicts that the emotional response in the trolley dilemma is lower than in the footbridge dilemma and cognitive control generally overrules the non-utilitarian emotional response. In contrast, the emotional response in the footbridge dilemma is much higher (reaches threshold) and the cognitive control does not overrule this response.

The situations described in moral-personal dilemmas are more emotionally salient than dilemmas in the non-moral and moral-impersonal dilemmas. The participant's decision might lead to serious bodily harm in another person, which will lead to feelings of empathy. Participants may also reason about the dilemma from the viewpoint of another person described in the dilemma, i.e., they use a TOM. Both feelings of empathy and TOM are related to activity in TPJ (Saxe, 2006; Young et al., 2010). Our results show an important role of the TPJ in moral judgment and are therefore in line with earlier findings. Experiments using fMRI (Greene et al., 2004), have found the cingulate cortex to be involved in moral judgment. In earlier studies, the cingulate cortex was found to be involved in the emotional response (Greene et al., 2001; Moll et al., 2005). Since the moral-personal dilemmas are more emotionally salient, the higher activity observed in the moral-personal condition is consistent with this. Another area that is conjectured to be associated with the emotional response is the temporal cortex (Heekeren et al., 2005; Moll et al., 2005).

In the chronometric TMS experiment we aimed to reveal the brain regions that are involved for distinct aspects of the moral decision-making process. Stimulation of the right DLPFC and right TPJ does not influence the reaction time or response in the control task (non-moral judgment) in a time-specific or time-unspecific manner. Therefore, any effect observed in the two experimental TMS sites (right DLPFC and right TPJ) can reasonably be assumed to be caused by the magnetic neural stimulation, and not by any general or time-specific effect of things such as discomfort, the clicking noise of the TMS coil, or startling effect of the TMS pulses itself. The statistical analysis of the responses after magnetic stimulation of the right DLPFC and right TPJ shows two interesting effects: (1) A time-specific double-dissociation of DLPFC and TPJ when looking at the difference between moral personal and moral impersonal dilemmas; and (2) a lower feeling of regret after the decision when TMS was applied over DLPFC when compared to application over TPJ.

First, there is a time-specific effect of TMS of the right DLPFC and right TPJ on moral decision-making. TMS over the right DLPFC and the right TPJ in moral judgment leads to a time-specific double dissociation in the roles of these brain areas. When stimulating the right DLPFC 2.5 s after onset of the question, the decision outcome in the moral-personal judgment condition becomes less utilitarian (see Figure 3A). The non-utilitarian decisions are associated with the decisions based on the initial emotional response. According to the dual-process theory, the DLPFC is involved in cognitive control over the initial emotional response. After TMS over the right DLPFC, behavior changes in such a way that is consistent with less cognitive control over the emotional response. If we consider the footbridge dilemma, the participant makes an emotional decision and is less likely to push a person off a bridge to save the lives of five others after TMS over the right DLPFC. When stimulating the right TPJ 2.5 s after onset of the question, the decision outcome in the moral-impersonal condition becomes less utilitarian (see Figure 3B). Although the moral-impersonal dilemmas do in itself elicit a weaker emotional response when compared to moral-personal dilemmas (Greene et al., 2001, 2004), the decision outcome of the moral-impersonal dilemmas changes in such a way that TMS seems to “boost” the emotional response in these dilemmas. If we consider the trolley dilemma, TMS of the right TPJ makes the participant less likely to turn the switch and change the path of the train to save the lives of five others at the expense of one.

Our main statistical analysis showed a double dissociation between the results of the DLPFC and TPJ. However, although this double dissociation seems to be driven by the results obtained in both the DLPFC as well as in the TPJ condition, we would like to point out that the *post-hoc* statistical analysis also shows that the results for the DLPFC are statistically more reliable than those for the TPJ.

Second, after making their non-moral, moral-impersonal, and moral-personal decision, participants were asked to evaluate the decision. Effects of TMS on the evaluation questions can be caused by either a direct TMS effect on the evaluation, or, more likely, an indirect effect on the evaluation that is caused by changing the decision process with TMS. We observed that, although we change the participants' moral judgment in a time-specific way, in hindsight, they do not change their response, they feel confident about their judgment, and they take full responsibility for their actions. The feelings of regret are influenced by the magnetic stimulation of the DLPFC. After right DLPFC stimulation, participants show less feelings of regret than after magnetic stimulation of the right TPJ. This last finding indicates that the right DLPFC is involved in evaluating the outcome of the decision process.

This experiment adds to the evidence of a critical role of right DLPFC and right TPJ in moral decision-making. Further experiments are needed to further enhance our understanding of how moral decision-making is accomplished in the human brain, and what specific aspects and cognitive processes are contributed to by the right DLPFC and right TPJ. Beyond such considerations, the current study is somewhat limited in statistical power because of the chronometric study design that includes many levels of the tested variables. Furthermore, the number of study participants is relatively small and in any case, it would be interesting to examine a larger subject pool to increase the generalizability of our findings. Such future studies should consider the effects of diverse cultural and ethnic backgrounds, as well as age, gender, education level, etc. Follow-up studies of our results are thus important to offer a replication and allow for further rigorous statistical testing of our findings, strengthen our conclusions, and allow examination of various individual factors and inter-subject variability. Furthermore, given that our frontal TMS target area does not exactly overlap with the coordinates of the frontal areas reported by Greene et al. (see for example Greene et al., 2001, 2004), it could be the case that there are several sub regions within the frontal cortex which are involved in moral decision making. Future research could aim to find out how these parts of the frontal cortex are involved in moral decision making and what their specific roles are in various moral cognition tasks.

In conclusion, there is a double dissociation between the DLPFC and TPJ in moral decision-making. Our results are in accordance with the dual-process theory, which distinguishes an initial emotional response and a more elaborative cognitive reasoning that can both influence the decision outcome. By influencing either the emotional response or the reasoning process in an online event-related TMS paradigm, we showed that the outcome of the moral decision could be influenced. Whereas disruption of the DLPFC during the decision makes the outcome of moral-personal judgment becomes less affirmative, disruption of TPJ affects moral-impersonal conditions. Disrupting activity in the DLPFC lead to lower feelings of regret after the decision, indicating that the DLPFC is involved in the evaluation of the decision.

## Author contributions

Danique Jeurissen, Alexander T. Sack, Alard Roebroeck, and Alvaro Pascual-Leone designed experiments, Danique Jeurissen and Brian E. Russ performed TMS experiment, Danique Jeurissen, Alexander T. Sack, Alard Roebroeck, Brian E. Russ, and Alvaro Pascual-Leone analyzed data and wrote the manuscript.

### Conflict of interest statement

The authors declare that the research was conducted in the absence of any commercial or financial relationships that could be construed as a potential conflict of interest.
